# The Prevalence of Violence Against Healthcare Workers in Trinidad and Tobago

**DOI:** 10.7759/cureus.58182

**Published:** 2024-04-13

**Authors:** Darren Dookeeram, Hariharan Seetharaman, Lake Taylor, Cherelle Stoute, Takiyah Toppin, Cassy Thomas, Jakeilia Trim, Kirtesha Thomas, Sade Stoute, Kanisha Caton

**Affiliations:** 1 School of Pharmacy, The University of the West Indies, St. Augustine, TTO; 2 Department of Emergency Medicine, Eastern Regional Health Authority, Sangre Grande, TTO; 3 Intensive Care Unit, The University of the West Indies, St. Augustine, TTO; 4 Faculty of Medical Sciences, The University of the West Indies, St. Augustine, TTO

**Keywords:** employee safety, healthcare administration, public health, healthcare workers, violence

## Abstract

Background:Abuse of healthcare workers (HCWs) and lack of public trust threaten the foundation of the physician-patient relationship. This growing global problem creates an even more difficult professional environment and hinders the delivery of high-quality clinical care.

Objective: The primary aim was to determine the prevalence of violence against Trinbagonian HCWs in the public sector. Secondary objectives included determining risk factors for violence and mistrust between the public and providers.

Method: A cross-sectional analysis of 434 HCWs in the public sector of Trinidad and Tobago was conducted using a modified World Health Organization (WHO) data collection tool, distributed via social media and administrative emails, and snowballed for two months. Fifteen semi-structured interviews were conducted regarding trust in the healthcare system with patients selected from communities.

Results: Of the 434 respondents, 45.2% experienced violence and 75.8% witnessed violence against HCWs in the past two years. Verbal abuse (41.5%) was most common. Perpetrators were patients (42.2%) and patients’ relatives (35.5%). Chi-square analysis highlighted that HCWs with the highest probability of being abused were aged 25-39 (63.8%), had two to five years of work experience (24.9%), specialized in emergency and internal medicine (48.6%), and cared for psychiatric and physically disabled patients (p-value < 0.001). HCWs believed the threat of violence negatively impacted performance (64.5%), and further action was necessary for mitigation (86.4%). Patients interviewed doubted physicians' altruism and competence (80%) and honesty (53.3%), expressed mistrust in their physician (46.7%), and cited poor infrastructure/management (66.7%) and dissatisfaction with care (60.0%) as factors that contributed to violence.

Conclusion: Analysis revealed that violence against Trinbagonian HCWs in the public sector deteriorated patient experience and adversely affected psychological well-being, efficiency, and job satisfaction. Results suggested mistrust of HCWs by the population. Interventions should be instituted to support at-risk HCWs and educate the public to avoid recurrence.

## Introduction

In 2020, the World Medical Association declared violence against Healthcare Workers (HCWs) an “International emergency that undermines the very foundations of health systems and impacts critically on patient’s health” [1]. The literature reflects that violence in the workplace for HCWs is a long-standing and multifactorial challenge to healthcare systems across the globe with a staggering 62% prevalence of physical violence [2]. The literature further details a myriad of factors that make this phenomenon more likely, including but not limited to understaffing, inadequate security, long waiting times, lack of privacy, and anger from patients and families [3].

The global experience of HCWs who have been the victims of violence reports a spectrum of injustices ranging from verbal, physical, sexual, and psychological [4]. Furthermore, the perpetrators of these acts are usually patients, patient’s relatives, and visitors [5]. Physicians, nurses, and auxiliary HCWs are often antagonized and intimidated by patients, patient’s relatives, and visitors [6]. Despite the high prevalence of this problem, there remains hesitation towards reporting which results in low levels of justice for HCWs [7].

The existing literature in the Caribbean is suggestive that a similarly high prevalence exists in Barbados [8] and Jamaica [9]. There is a paucity of studies in this area in Trinidad and Tobago on this subject matter; this study therefore serves to bridge a gap as it aims to quantitatively and qualitatively assess the prevalence of violence in the public healthcare system of Trinidad and Tobago. This is likely to be reflective of many other post-colonial islands in the Caribbean which have a predominantly publicly funded healthcare system and a smaller but well-established insurance and out-of-pocket private system.

This article was previously presented as a meeting abstract at the World Congress of the World Association for Disaster and Emergency Medicine in Killarney Ireland, in May 2023.

## Materials and methods

The primary research question was “What is the Prevalence of Violence against Trinidadian HCWs in the public sector?” A secondary research question was “Is there a link between the prevalence of Trinidadian healthcare workplace violence and the level of public trust?”

The setting was in the first instance a cross-section quantitative study of public HCWs and in the second instance a semi-structured interview of volunteers from varying geographical areas in Trinidad and Tobago. Institutional approval for this study was obtained from the University of the West Indies and the Eastern Regional Health Authority in Trinidad and Tobago (REFERENCE CREC-SA.1252/11/2021). The data were collected from HCWs through the use of Google Forms, an online data collection platform. HCWs from an approved RHA were recruited via social media and administrative emails.

In the first phase, questionnaires to the HCWs were allowed to snowball over the period of January 31 to March 15, 2022 to attain a target sample size of 369. The questions asked to the HCWs were derived from the World Health Organization’s violence tool “Workplace Violence in the Health Sector.” In the second phase of the study, 15 interviewees were conveniently chosen based on their location and utilization of the public healthcare facilities. These interviews were conducted virtually on Google Meet, an online conference platform. The interviewees' responses were used to evaluate the level of trust between the public and HCWs and were derived de novo by the researchers.

Karn et al. in a 2021 publication found that 60% of HCWs had been exposed to some type of violence [2]; using this prevalence the sample size for HCWs to achieve statistical significance was 369 in Phase 1 of the study. For patient feedback, three patients who utilize the public healthcare system were selected from each of the semi-autonomous regional health authorities for interview. Only HCWs in Phase 1 and members of the public who have utilized public healthcare facilities in Phase 2, who voluntarily consented to participate were included in the study. This was done via a consent form which noted that participants were free to decide whether to submit the questionnaire or refrain from answering specific questions. Hence, the study automatically excluded those unwilling to participate and persons who did not want to or were unable to consent (under the age of 18). In adherence to ethical guidelines, approval was received before data collection, participants were all 18 years and over and were required to give informed consent. Lastly, no identifiable information was captured, and the participants’ responses were stored on a secure medium where only investigators had access.

In the first phase of the study, data collection from the HCWs was facilitated via online questionnaires adapted from WHO’s validated assessment tool entitled, “Workplace Violence in the Health Sector.” Google Forms was selected as the platform to deliver the questionnaires to the HCWs. The questionnaire consisted of 33 questions which included demographic, rating scale multiple-choice questions, and one open-ended question inclusively. There was no direct contact with the researchers and all responses were not associated with any unique identifiers on the platform. This allowed for the collection of concise information pertaining to the study and was necessary for answering the chosen research questions.

In the second phase of the study, a series of standardized semi-structured interviews was conducted via Google Meet with patients selected from communities pertaining to trust in the healthcare system and providers. All data obtained from interviewees were collected onto a secure Google spreadsheet file. The data was analyzed using Statistical Package for the Social Sciences (SPSS) software (IBM Corp., Armonk, NY) - version 28.0.1.0 (142) - which allowed for descriptive analysis. The software was also used to perform comparative analysis via chi-squared tests.

## Results

There were 434 respondents to the online survey. Participants were primarily female (66.6%), of East Indian descent (50.2%), non-immigrants (88.9%) currently working as physicians (64.1%), mainly in Emergency medicine (24.9%) and Internal medicine (23.7%). Most respondents worked with more than five colleagues (58.1%) and cared for more than 15 patients daily (51.8%) which included males and females (87.1%). The respondents cared for patients of varying ages and had various years of experience.

As depicted in Table 1, 75.8% of participants witnessed WPV in the last two years, while 45.2% were victims of said violence. Verbal abuse was the most common form, and the most frequently observed perpetrators were patients (34.6%) and patients’ relatives (38.7%). Of those that reported, 22.8% were not satisfied with resolution attempts and 19.8% did not report as it was deemed useless. Additionally, 64.5% noted that the threat of violence had either a moderate/strong impact on their ability to perform duties.

**Table 1:** Workplace violence characteristics by number and percentages

Characteristic	Number (N)	Percentage (%)
Witnessed violence in last two years		
Yes	329	75.8
No	91	21
No response	14	3.2
Experienced violence in last two years		
Yes	196	45.2
No	224	51.6
No response	14	3.2
Observed perpetrators		
Patient/ client	150	34.6
Relative of patient	168	38.7
Colleague/ staff member	16	3.7
Supervisor/ senior	9	2.1
Member of general public	18	4.1
N/A	51	11.8
No response	22	5.1
Frequency of workplace violence		
Very common	54	12.4
Moderately common	196	45.2
Not common	168	38.7
No response	16	3.7
Types of violence experienced		
Physical	26	6.0
Sexual	1	0.2
Verbal	180	41.5
N/A	158	36.4
Other	1	0.2
No response	68	15.7
Perpetrators of workplace violence		
Patient/Client	183	42.2
Relative of patient	154	35.5
Colleague/ staff member	42	9.7
Member of general public	30	6.9
Has the incident been dealt with		
Yes	93	21.4
No	145	33.4
No response	196	45.2
Who was the incident reported to		
Management/ employer	49	11.3
Union	130	30.0
Colleagues	101	23.3
Friend/ family member	49	11.3
Police	25	5.8
Other	9	2.1
Not applicable	158	36.4
Do you think additional action is required		
Yes	375	86.4
No	42	9.7
No response	17	3.9

As shown in Table 2, 34.1% respondents indicated that there should be an increase in security, 7.8% stated that conflict resolution training should be given, while 21.4% believe that harsher penalties should be enforced for perpetrators. Lastly, 35.7% had other non-specific recommendations.

**Table 2:** Recommendations given by HCWs to mitigate workplace violence

Recommendations	Number (N)	Percentage (%)
Increased security (properly trained personnel, security cameras and screening on entry)	148	34.1
Conflict management/ resolution training	34	7.8
Harsh penalties/ charge perpetrators	93	21.4
Improved and greater support from management	24	5.5
Bring awareness/ no tolerance policy to abuse	35	8.1
Other	96	22.1

As depicted in Table 3, most interviewees did not believe their physician was extremely thoughtful and careful (80%) or totally honest in revealing all treatment options for a condition (53%) and over 45% of respondents noted they did not have complete trust in their physician.

**Table 3:** Trust between the public and healthcare workers via focus interviews

Questions	Strongly Agree (%)	Agree (%)	Disagree (%)	Strongly Disagree (%)	No Opinion (%)
Sometimes your doctor cares more about what is convenient for him/ her than your medical needs	3 (20%)	3 (20%)	5 (33%)	3 (20%)	1 (7%)
Your doctor is extremely thorough and careful	1 (7%)	0 (0%)	3 (20%)	9 (60%)	2 (13%)
You completely trust your doctor’s decisions about which medical treatments are best for you	1 (7%)	3 (20%)	2 (13%)	3 (20%)	6 (40%)
Your doctor is totally honest in telling you about all of the different treatment options available for your condition	1 (7%)	3 (20%)	2 (13%)	3 (20%)	6 (40%)
All in all, you have complete trust in your doctor	1 (7%)	3 (20%)	0 (0%)	7 (47%)	4 (27%)
Sometimes doctors are more about what is convenient for them than about their patients’ medical needs	0 (0%)	4 (27%)	4 (27%)	3 (20%)	4 (27%)
Doctors are extremely thorough and careful	1 (7%)	2 (13%)	5 (33.3%)	5 (33.3%)	2 (13%)
You completely trust doctors’ decisions about which medical treatments are best	0 (0%)	5 (33%)	0 (0%)	2 (13%)	8 (52%)
A doctor would never mislead you about anything	2 (13%)	5 (33%)	4 (27%)	4 (26.7%)	0 (0%)
All in all, you trust doctors completely	0 (0%)	5 (33%)	1 (7%)	6 (40%)	3 (20%)
The Health Authorities are good at what they do	1 (7%)	2 (13%)	4 (27%)	7 (47%)	1 (6.7%)
When needed the Health Authority will provide everything that is needed	2 (13%)	7 (47%)	2 (13%)	2 (13%)	2 (13%)
When questioned the Health Authorities are honest with their answers	1 (7%)	5 (33%)	4 (27%)	3 (20%)	2 (13%)
The Health Authorities will pay for everything it is supposed to, including treatment that is expensive	3 (20%)	3 (20%)	2 (13%)	5 (33%)	2 (13%)
Public hospital only care about keeping medical costs down and not what is needed for my health	1 (7%)	2 (13%)	3 (20%)	6 (40%)	3 (20%)
Public hospitals provide the highest quality in medical care	2 (13%)	6 (40%)	2 (13%)	5 (33%)	0 (0%)
When treating my medical problems, public hospitals put my medical needs above all other considerations, including costs	1 (7%)	5 (33%)	3 (20%)	2 (13%)	4 (27%)
COVID-19 Public services have become more difficult to access	0 (0%)	1 (7%)	0 (0%)	8 (53%)	6 (40%)
COVID-19 Public HCWs spend less time with me now than before	1 (7%)	2 (13%)	4 (27%)	6 (40%)	2 (13%)
All of my needs are taken care of when I attend public hospitals	1 (7%)	3 (20%)	3 (20%)	1 (13%)	6 (40%)
Do you agree with the WMA statement “Violence against health personnel is an international emergency that undermines the very foundation of health systems and impacts critically on patients’ health”	0 (0%)	2 (13%)	1 (6%)	11 (73%)	1 (7%)

Most interviewees cited poor infrastructure/management (66.7%) and patient dissatisfaction with care (60.0%) as factors that contribute to violence against HCWs (Table 4).

**Table 4:** Factors contributing to violence against HCWs from a patient's point of view

Factors	Frequency (%)
Poor infrastructure and management (lack of healthcare resources and staff)	10 (66.7%)
Dissatisfaction with service (decreased interaction time, poor communication and increased mistakes)	9 (60%)
Healthcare workers are unprofessional (abusive/biased/ not patient centred)	7 (46.7%)
Overworked / stressed HCWs with long shifts	2 (13.3%)
Emotionally charged patients (angry, frustrated, anxious) in a stressful environment	5 (33.3%)
No policies in place to protect HCWs and reprimand abusive patients	2 (13.3%)
Apathetic, unkind & desensitised HCWs with no passion	4 (26.7%)
Other	5 (33.3%)

As demonstrated in Table 5, Chi-squared testing for significance showed that younger age and field of employment were statistically associated. Younger HCWs were at higher risk and those employed in Emergency medicine and Internal Medicine were at higher risk.

**Table 5:** Chi squared analysis for significant relationships with violence against HCWs

Characteristic of HCW	P-value associated with being a victim
Gender	0.466
Ethnicity	0.542
Decreasing Age	<0.001
Nationality	0.886
Category of employment	0.802
Field of employment	<0.001

When asked “To what extent does workplace violence negatively impact performance as a healthcare professional?,” 32.7% responded moderate impact and 31.8% responded strong impact. When asked about mood during working hours taking into consideration the risk of experiencing violence, 43.5% reported feeling anxious, 14.1% reported feeling angry and 6.2% reported feeling fearful.

## Discussion

Trinidad and Tobago is a Small Island Developing State in the Caribbean and in many ways is reflective of the socio-political post-colonial health systems of the region and among many other similar states. The health system constitutes a central governance structure with multiple semi-autonomous regional health authorities that are responsible for the operational arms of service, including security [10]. The results of this study demonstrated that the study population experienced a high level of violence against HCWs, and while it may not have been as high as the 60% it is still alarming that nearly half of the study population claimed to be victims.

With 45.2% reporting being victims, and 75.8% having witnessed violence in the two years prior to the study, there is a clear need to address this issue which poses a foundational threat to the efficiency of the medical system. 64.5% of HCWs indicated that the experience had a strong to moderate impact on their performance and 86.4% concurred that additional measures need to be implemented to mitigate the impact. These sentiments are reinforced in a 2015 article published by local news media which voiced the distress and frustration felt among Trinidad and Tobago’s HCWs due to the inadequacies of the public system to protect them from the physical and verbal abuse that has been disregarded over the years [11]. It has long been recognized that there are serious direct and indirect costs to violence against HCWs and the profound psychological toll that it exerts [12]. There is therefore much to be considered through this study with respect to the rationalization and validity of the experiences of the Trinidad and Tobago HCW population.

Consistent with the literature, it was expected that the most common type of abuse would be verbal (41.5%) then physical (6.0%). Additionally, the investigation of the main perpetrators of these types of abuse exposed patients (34.6%) and patient’s relatives (38.7%). The nature of work in healthcare requires consistent interaction with persons who may be unpredictable and emotionally labile combined with sometimes challenging systemic factors; these are cited as significant risk factors by the Agency for Healthcare Research and Quality (AHRQ) [13].

From the HCW's point of view, the results indicated various reasons for the patients' forwardness in abusing HCWs. The shortage of security presence within the workplace (34.1%) was the leading perception that warranted disorderly behavior. Secondly, the lack of implementation of harsh penalties (21.4%) means that abuse will continue unless appropriately addressed. Thirdly, 8.1% of the HCWs indicated the need for increased public awareness/signage on the institution’s no-tolerance to violence against HCWs, notwithstanding the 7.8% who expressed that HCWs need conflict resolution training to help defuse potential conflicts. Lastly, 5.5% of HCWs indicated that more support is required from management since 33.4% stated no action was taken when they were abused, as shown in Table 1. This was reflected by 19.8% of the HCWs who stated that it was “useless” to report; 5.3% who were unaware of who to report the incident to and 9.7% noting that the process was too tedious. These findings were consistent with the Centers for Disease Control and Prevention and strongly suggest that healthcare administrators should be more proactive in the prevention of circumstances that may lead to this occurrence [14].

A deeper look at the key determinants that made a selective category of HCWs a target resulted in a few discoveries. Firstly, HCWs ages 25-39 were most at risk and accounted for more than 50% of WPV victims. This can be due to this age category being predominantly junior staff/interns who may lack fluidity and sometimes legitimacy within the field. The next at-risk group of HCWs were those with two to five years of experience, which is connected to the aforementioned reason. And, unsurprisingly, emergency and internal medicine specialty HCWs were most targeted. Lastly, HCWs for psychiatric and physically disabled patients had a significantly greater probability of being abused. The impaired judgment of these patients can explain this expected outcome. However, gender and ethnicity unexpectedly proved not to be a significant factor related to workplace violence. These risk factors should also be taken into consideration by administrators who are responsible for security allocations within healthcare facilities [14].

The impact that violence against HCWs is profound with the majority of persons experiencing strong emotional disturbance that could potentially affect their function and productivity. It is imperative that support be provided for these victims since an impaired HCW is unlikely to provide the highest quality of care to patients. Furthermore, it remains the responsibility of the employer to ensure that a safe working environment is provided for employees. Given that there are clear risk factors, additional measures should be put into place to ensure that vulnerable groups are given the security support that is necessary. Additionally, the HCWs who are victims should be assured of having psychological support and appropriate feedback for any complaints that are made to the administration. 

Analysis of patient perspectives revealed that the majority of them strongly disagreed (47.7%) when asked if they trusted their doctors completely. 60% of respondents strongly disagreed that their doctor is extremely thorough and careful. Additionally, 20% expressed that their doctor sometimes cares more about what is convenient for him/her than about their medical needs. Furthermore, the majority of patients (53.3%) indicated that since the COVID-19 pandemic, access to public healthcare has not become difficult, 66.7% expressed that they still get their usual time spent with physicians prior to the pandemic.

Despite a prominent lack of trust in public sector HCWs, when asked the reasons for violence against HCWs, interviewees mentioned factors mainly; poor infrastructure and management (66.7%) and dissatisfaction with service due to decreased interaction times or increased medical errors (60%) were the main responses. Although the majority of patients lack trust in their healthcare providers, there did not appear to be a link between violence against HCWs and a shift in trust between themselves and members of the public. These findings underscore the importance of building a trusting relationship between patients and HCWs which should be fostered by employers [15].

The limitations of the methodology included that HCWs who were not able/comfortable with smartphone devices were less likely to participate in the study. This may have created a bias in decreasing enrollment of those in lower socioeconomic brackets or less technology savvy. The method of snowballing in data collection also limited control of the cross-section meaning that subsets within HCWs could not be deconstructed for more detailed analysis.

## Conclusions

This study revealed preliminary evidence of the prevalence of violence against HCWs in the public sector of Trinidad and Tobago. Moreover, components such as age, years of experience, specialty, and the type of patient mostly encountered, were found to be determinants of workplace violence. Findings suggested that continued acts of violence; verbal abuse being the main type, were due to a lack of patient awareness of the institution’s “no violence policy”, insufficient security, and lack of support from management. This has the potential to affect workplace performance and job satisfaction. Health system interventions should be instituted to support at-risk HCWs as well as educate the public to avoid recurrence. Successful resolution of this challenge pivots upon the evolution of healthcare systems to understand and meet the changing needs of patients.

Employers should regard incident reports as matters of grave importance as the majority of HCWs who experienced violence stated that the matter was not dealt with. Strategies such as implementing harsher charges/penalties toward perpetrators should be enforced by managed to prevent these violent displays. Support such as counseling or time off should also be rendered to victims after such traumatic incidents. Additionally, awareness of workplace violence is important and conflict resolution training is integral in reducing the prevalence of such acts. Increasing security would not only make HCWs feel safe but can act as a deterrent for potential offenders. Lastly, the practitioner: patient ratio should be amended to avoid burnout which may act as a catalyst for violent acts. Patients should be given alternative and meaningful recourse to vent their concerns and problems with the healthcare system.
